# The complete chloroplast genome sequence of *Ventilago leiocarpa* Benth

**DOI:** 10.1080/23802359.2020.1861559

**Published:** 2021-03-11

**Authors:** Xiang Lu, Qiuxiang Luo, Yiming Qin, Qiwei Yan, Song Guo

**Affiliations:** a College of Food and Biochemical Engineering, Guangxi Science and Technology Normal University, Guangxi Laibin, PR China; b Key Laboratory for Research and Development of Characteristic Yao Medicine Resources, Guangxi Science and Technology Normal University, Guangxi Laibin, PR China; c Yao Medical Hospital of Jinxiu Yao Autonomous County, Guangxi Laibin, PR China

**Keywords:** *Ventilago leiocarpa* Benth, chloroplast genome, Rhamnaceae

## Abstract

*Ventilago leiocarpa* Benth. is an important medicinal and edible plant. The complete chloroplast genome of *V. leiocarpa* Benth. was assembled and annotated. In this study, the chloroplast genome of *V. leiocarpa* Benth. was a circular form of 161,880 bp in length. The genome presented a typical quadripartite structure consisting of a pair of inverted repeats (IRa and IRb) of 26,357 bp separated by a large single copy (LSC) region of 90,056 bp and a small single copy (SSC) region of 19,129 bp. The genome contained a set of 127 genes, including 82 protein-coding genes, 37 tRNA genes, and 8 rRNA genes. Phylogenetic analysis showed that *V. leiocarpa* Benth. closely related to *Rhamnus taquetii*, which beyond to Rhamnaceae.

*Ventilago leiocarpa* Benth. belongs to the Rhamnaceae family. The sterm of *V. leiocarpa* Benth., ‘Zi-jiu-niu’ in Yao medicine of China, was offen used as an analgesic and for the treatment of cough, rheumatism, contused wounds, etc. (Lin et al. 1996, 2001; Zhou et al. 2001). *V. leiocarpa* Benth is rich in quinine compounds, such as emodin, physcion, ventilagolin, and ventiloquinone-I (Hu et al. 2020). The extract from the stem of *V. leiocarpa* Benth had anti-inflammatory and hepatoprotective properties (Chang et al. 1996; Hu et al. 2020). It is widely used in Guangxi Jinxiu, China. However, the phylogenetic relationships of the *V. leiocarpa* Benth. have never been well tested. In this study, Illumina technology was applied to sequence, assemble and annotate the whole chloroplast genome of *V. leiocarpa* Benth.

The fresh leaves of *V. leiocarpa* Benth. were collected from Jinxiu Yao Autonomous County, Guangxi Province, China (N:24°11′5.74″, E:109°59′59.57) and the specimen was stored at Yao Medical Hospital of Jinxiu Yao Autonomous County Herbarium (ZJN202006). Five pieces of young leaves were used in this experiment. Total DNA was extracted by using a DNeasy Plant Mini Kit (QIAGEN, Hilden, Germany). The high-quality DNA was sheared to the size of 300 bp for the shotgun library construction and the Genome was sequenced using Illumina Novaseq PE150 platform (Illumina Inc, San Diego), and 150 bp paired-end reads were generated.Low-quality reads and adapters were removed by the FastQC software (Andrews 2010). Paired end clean reads were combined and a total of 17,588,694 sequences were obtained for chloroplast genome assembly. Using the assembly method of Hahn et al.(Bernt et al. 2013), the reference sequence was selected from the published NCBI *Berchemia berchemiifolia* chloroplast genome (GenBank accession number: NC_037477). The annotation was mainly carried out by comparing the chloroplast genomes of related species, and the annotation results were confirmed and modified by CPGAVAS online tool (Zuo et al. 2017), and the reference genomes used for annotation is also *B. berchemiifolia* chloroplast genome (NC_037477). The annotated genomic sequence was registered into GenBank with an accession number (MT974496).

The complete chloroplast of *V. leiocarpa* Benth. was 1,61,880 bp in length, consisting of a large single copy region (LSC, 90,056 bp), a small single copy region (SSC, 19,129 bp), and two inverted repeat regions (Ira and Irb, 26,357 bp). The overall GC content of the cp genome was 37.04%, while that of IR regions (42.9%) higher than that in LSC (34.9%) and SSC regions (31.3%). A total of 127 unique genes were identified from the chloroplast genome of *V. leiocarpa* Benth., among which are 82 protein coding genes, 37 tRNA genes and 8 rRNA genes. The intron-exon structure analysis indicated that 18 genes have introns, among which trnK-UUU, rps16, rpoC1, atpF, trnG-GCC, trnL-UAA, trnV-UAC, petB, petD, rpl16, rpl2, ndhB, trnl-GAU, trnA-UGC, and ndhA have one intron, while ycf3, rps12, and clpP have two introns.

Rhamniaceae is a kind of dicotyledonous plants, including Rhamnaceae, Vitaceae and Leeaceae. To analyze the phylogenetic relationship of *V. leiocarpa* Benth., the complete chloroplast genome sequences of 10 Rhamniaceae family species from the NCBI GenBank database were downloaded. The phylogenetic tree was generated based on whole chloroplast genome sequences (Shen et al. 2020). The 11 complete chloroplast sequences were aligned by the MAFFT version 7.450 software (Katoh and Standley 2013). Phylogenetic analysis was conducted based on maximum likelihood (ML) analyses implemented in IQ-TREE 1.5.5 (Nguyen et al. 2015) under the TVM + F + R2 nucleotide substitution model (1000 bootstrap replicates), which was selected by ModelFinder (Kalyaanamoorthy et al. 2017; Ji et al. 2020).

The phylogenetic trees were analyzed with MEGA6 software (Koichiro et al. 2013) using maximum likelihood (ML) method (Bootstrap values were calculated out of 1000 replicates) (Yu et al. 2020). The phylogenetic analysis showed that *V. leiocarpa* Benth. closely related to *Rhamnus taquetii*, which beyond to Rhamnaceae. Our study will provide useful information on further clarifying the phylogenetic and evolutionary relationship in the Rhamnaceae (Figure 1).

**Figure 1.**
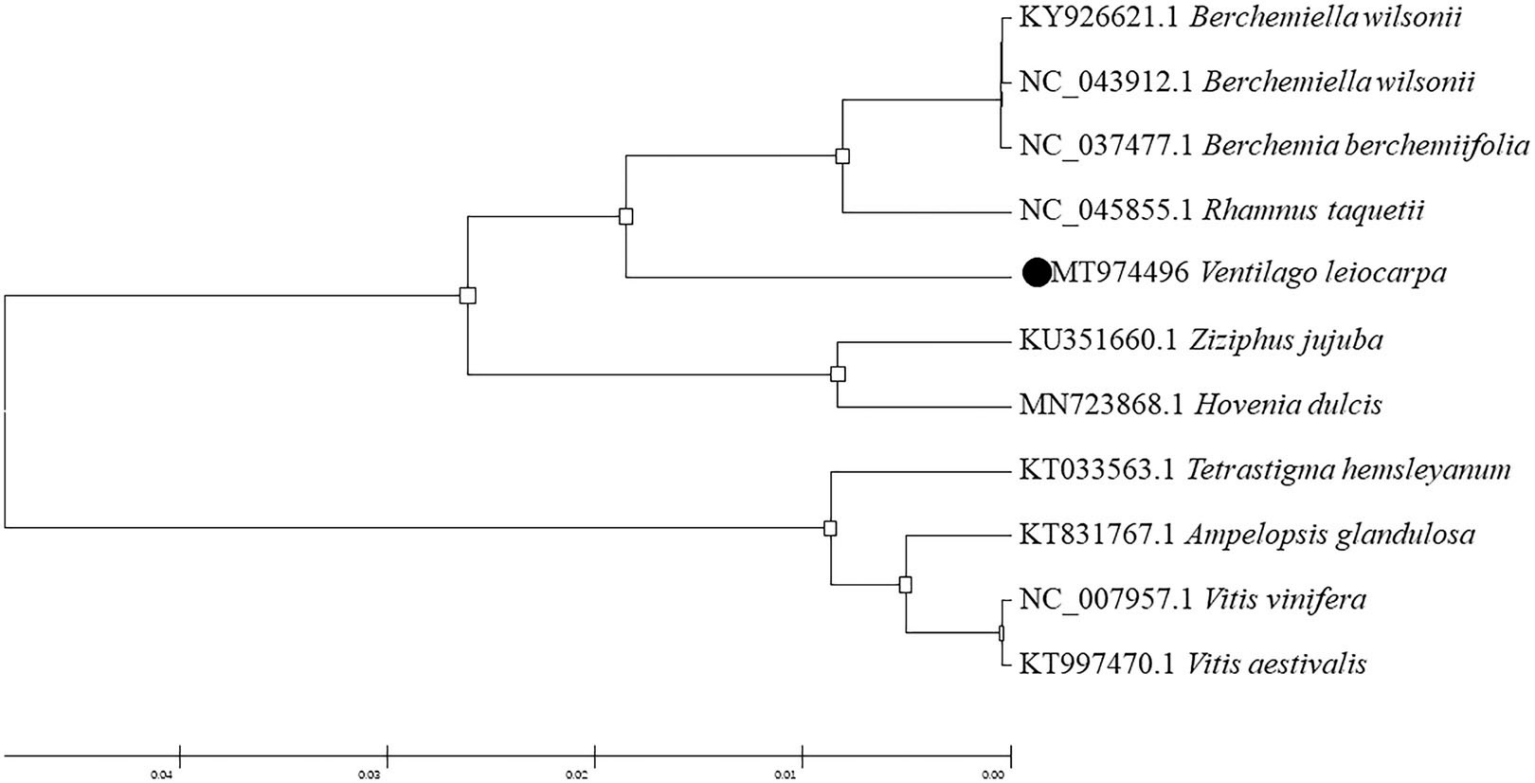
Phylogenetic placement of *V. leiocarpa* Benth. in the framework of Rhamniaceae resolved by maximum likelihood method based on the complete chloroplast genome.

## Data Availability

The data that support the findings of this study are available in NCBI SRA at https://www.ncbi.nlm.nih.gov/sra/PRJNA662837, reference number PRJNA662863.
